# A potential energy and mutual information based link prediction approach for bipartite networks

**DOI:** 10.1038/s41598-020-77364-9

**Published:** 2020-11-26

**Authors:** Purushottam Kumar, Dolly Sharma

**Affiliations:** Department of Computer Science and Engineering, Shiv Nadar University, Gautam Buddha Nagar, 201314 Uttar Pradesh India

**Keywords:** Computational science, Computer science

## Abstract

Link prediction in networks has applications in computer science, graph theory, biology, economics, etc. Link prediction is a very well studied problem. Out of all the different versions, link prediction for unipartite graphs has attracted most attention. In this work we focus on link prediction for bipartite graphs that is based on two very important concepts—potential energy and mutual information. In the three step approach; first the bipartite graph is converted into a unipartite graph with the help of a weighted projection, next the potential energy and mutual information between each node pair in the projected graph is computed. Finally, we present Potential Energy-Mutual Information based similarity metric which helps in prediction of potential links. To evaluate the performance of the proposed algorithm four similarity metrics, namely AUC, Precision, Prediction-power and Precision@K were calculated and compared with eleven baseline algorithms. The Experimental results show that the proposed method outperforms the baseline algorithms.

## Introduction

The applications of link prediction in real-world networks has been attracting the attention of researchers from various domains. The real world networks can be represented with the help of graphs, where nodes represent entities that are connected by edges. The edges represent associations or interactions between nodes. These networks are dynamic in nature, where new nodes and edges can be observed at future timestamps. Link prediction task aims to identify the most probable links that may appear in the near future. Some of the popular real-world applications of link prediction are: Prediction of hidden relationships between terrorists, e-commerce recommendation system, prediction of drug side effects, protein-protein interaction prediction, finding missing reactions in metabolic networks, predicting co-authorship. Link prediction problem takes as input a snapshot of the network and computes the likelihood of a connection between two unconnected vertices^1–3^. The algorithms not only predicts the addition of new edges, it also predicts the connections that may disappear from the network in future. Most of the link prediction algorithms have been designed for unipartite networks. The structure and properties of bipartite networks are different from unipartite ones. For this reason the algorithms that perform well for unipartite networks may not work well for bipartite networks. Link prediction on Bipartite networks have applications in Recommendation systems. Li et al.^4^ used applied link prediction to build recommender systems based on the concept of graph kernels. They generated random walks starting from a focal user-item pair in the graph kernel. Due to the fact that defining features in the complex graph is challenging, authors used a kernel-based machine learning framework that works on kernel function and this function defines the similarity between data instances. The authors performed testing on three real-world datasets and the it was observed that the proposed method outperformed the baseline algorithms. Kurt et al.^5^ also proposed a graph-based recommender system (SIMLP) that computed similarity between two nodes in a bipartite network. They assigned complex numbers as the weight of an edge connecting a node pair. SIMLP algorithm was tested on two datasets and the results show that it algorithm performed better than the complex number-based baseline algorithms.

We have used the terms graphs and networks interchangeably throughout the paper. The Link Prediction problem can formally be defined as follows.

*Problem statement* Given a bipartite graph *G*(*U*, *V*, *E*), where *U* and *V* are two disjoint and independent sets of vertices and *E* is set of edges at timestamp $T_1$. The link prediction algorithm aims to predict new links among nodes that can be observed at timestamp $T_2$, such that $T_2 > T_1$. These links may be due to one of the two reasons; either due to the formation of a new connection, or due to revival of an existing missing connection.

In this work, the authors make the following contributions in order to propose new algorithm for the above mentioned problem:

1. Two parameters have been introduced with respect to link prediction: potential energy and mutual information.
2. Potential Energy and Mutual Information based similarity metric (PMIS) proposed to compute the weight of the patterns.
3. The proposed algorithm works on each disconnected node pair in the network instead of the only candidate node pair.
4. The proposed algorithm evaluates on ten real world datasets and demonstrates superior performance of the proposed algorithm compared to baseline link prediction techniques.

Link prediction algorithms^6–10^ usually follow one of the following four approaches: Node Based link prediction, Neighbor-based link prediction, Path-based and Social Theory-based link prediction. Liben-Nowell et al.^1^ analysed various proximity measures and suggested proximity measures for best prediction results among node neighborhoods approach, path-based approach and Higher-level (meta) approaches. Common neighbors, Jaccard coefficient, Adamic/Adar coefficient are common neighbor based approaches. Triangle-closing model states that node-pairs with a high number of common neighbors try to form a triangle in the graph. Such node-pairs have a high probability of forming connections in the future. This concept does not apply to bipartite networks because of their distinct topological structure. Thus common neighbor based approaches can not apply directly on bipartite networks. Hasan et al.^11,12^ further extended the work of Liben-Nowell et al.^1^. Link prediction for bipartite networks has been addressed by many researchers^4,13–19^. Cannistraci et al.^20^ proposed an algorithm to target not only common neighbors and neighbor’s common neighbors but also to their connections structure. This was the first attempt of bipartite formulations of the Common Neighbor index. Further, the authors in^21^ used the concept of local community paradigm (LCP-theory) for link prediction and states that the cohort of CNs and their cross interactions form a local-community edge. The cross interactions between common neighbors are called local community edges or links. The LCP-based method presented in^21^ improves topological prediction in bipartite complex networks^13^ used the concept of projection and supervised learning for link prediction. They introduced three link prediction metrics for bipartite graphs and implemented these metrics on DBLP dataset.Baltakiene et al. in^22^ presented the concept of entropy and used the Bipartite Configuration Model (BiCM)^23^ as a score function for predicting links. As used in Statistical Mechanics, probability per graph can be derived by maximizing the Shannon entropy under the constraint of the degree sequence. So by using the concept of maximal entropy they significantly improved the performance of link prediction. Gao et al. used the concept of projection and CNP(candidate node pairs) for link prediction in the bipartite networks^24^. The authors first converted the bipartite graph into unipartite graph and then computed the CCNP (Connectivity of candidate node pairs) with the help of pattern weights for link prediction. They evaluated the performance of the algorithm on three datasets and experienced better results than baseline predictor(CN, Katz, ILP^18^) on AUC. Shakibian et al. proposed another similarity measure based on mutual information and meta-path in heterogeneous networks^25^. They presented a framework in which link entropy is characterized as a semantic measure for link prediction. To measure the effectiveness of the algorithm, authors compared it with different classes of link prediction algorithms namely, mainstream meta-path based link predictors, effective path-based homogeneous link predictor, and LCP-based link indicators. The authors analysed the performance of algorithm on DBLP network. In 2019 Serpil et al. used strengthened projection technique for link prediction in evolving bipartite graph^26^. They tried to predict link in large scale bipartite networks. Authors did link prediction in mainly two steps. In the first steps, they extracted potential link set and in the second step, they computed the prediction score of each potential link. For this purpose authors proposed a time aware proximity measure based on network evolution. For the result analysis, they used AUC and precision metrics. In the experiment, the authors compared his method with four baseline algorithms (AA, CN, JA, PA). And they found that his algorithm outperforms the baseline algorithm. It is often observed that the complete information about real-world complex networks is not available. Some examples of such networks are financial networks, social networks and biological networks. Cimini et al.^27^ presented an exhaustive review on statistical physics based approaches to predict statistically significant patterns in complex networks. They also addressed the reconstruction of network structure in the absence of complete information. Boguna et al.^28^ presented a review on network based approaches that very effectively identify both physical properties and mathematical properties that are fundamental to networks. They discussed three approaches and proposed interesting future directions. In the review they presented that in case of heterogeneous networks, the models based on hyperbolic space could be better than Euclidean space. In addition the hyperbolic space could be used for link prediction.

## Preliminaries

### Projection of bipartite graph

Projection of bipartite graphs can be used to convert it into unipartite graphs. For a given bipartite graph $G =( U,V,E)$, its U-projected graph can be represented as a unipartite graph $G_u =(U,E_u)$ in which $(A, B) \in E_u$ if *A* and *B* have at least one neighbor common in *G*. This means, $N(A) \cap N(B)\ne \phi$ and $E_u$ can be described as follows:

$$\begin{aligned} E_u= \{(A,B)\mid A,B \in U, \exists x \in V,x\in N(A)\cap N(B)\} \end{aligned}$$

**Figure 1:**
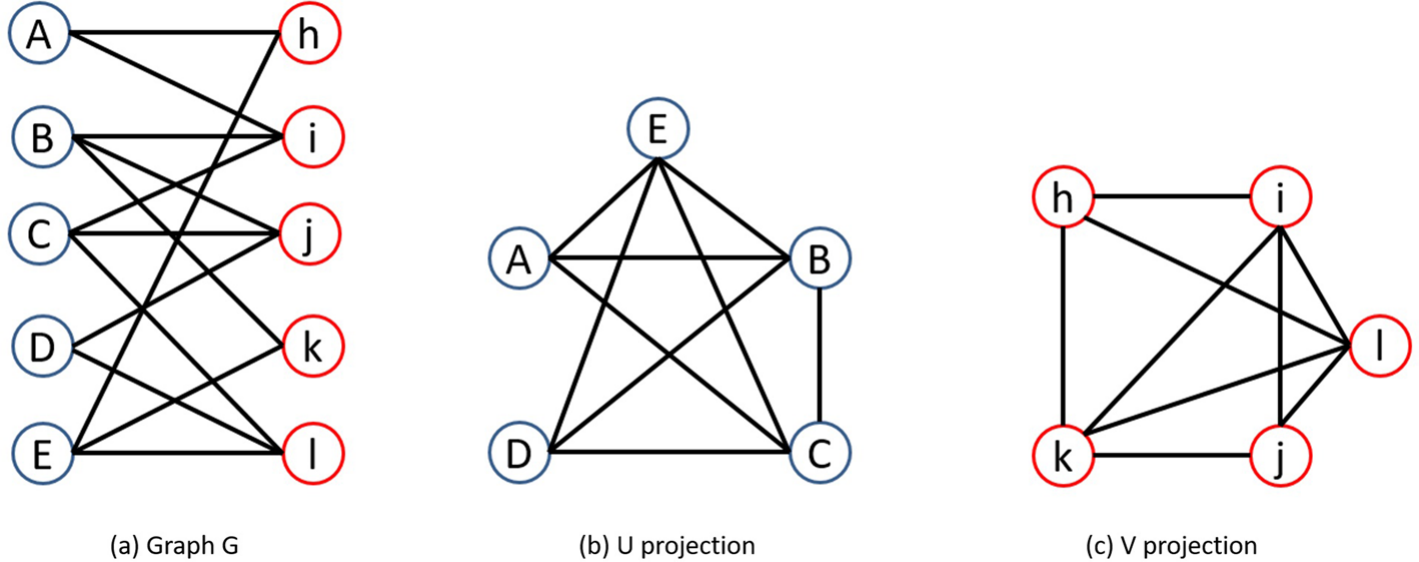
Projection of bipartite graph G.

Here two projections of the graph can be taken, one for *U* and another for *V*. V-projection can be defined for the graph similar to its U-projection. For a graph *G*, it’s V-projected graph will be $G_v=(V, E_v)$. For example Fig. 1a denotes the graph G where values of *U* and *V* are $\{A,B,C,D,E\}$ and $\{h,i,j,k,l\}$ respectively, Fig. 1b denotes their U-projection and Fig. 1c denotes the V-projection.

### Pattern and pattern covered by a Node pair

*Pattern* Suppose A and B are two nodes in a bipartite graph *G*(*U*, *V*, *E*) and $\{A, B\} \in U$. Then (*A*, *B*) forms a pattern if they have at least one common neighbor in *V*^24^. Therefore a link (A, B) will be available in the projected graph. We can also say that each link $(A,B) \in E_u$ in the projected graph represents a pattern in bipartite graph G.

*Pattern covered by a Node pair* Suppose (A, i) be a node pair in bipartite graph G and $G_u$ be the projected graph of G. For each node $C \in N_u (A) \cap N (i)$, we call {A, C} a pattern covered by node pair(A, i)^24^. A node pair may cover one or more patterns in the projected graph. Pattern covered by a node pair simply says that a similar edge has already existed in bipartite graph G. The more patterns a node pair covers, more are the chances that this node pair will be connected in the future. In this manner, the number of patterns secured by a node pair can be utilized to measure the likelihood of its edge presence.

### Mutual information for link prediction

Our proposed algorithm uses the concept of mutual information and potential energy for link prediction in the bipartite graph.

Self-information: “Let X be a random variable and x be an outcome of X with probability p(x). Then, the self-information of x quantifies the uncertainty of the outcome x and is defined as follows”^29^:

1 $$\begin{aligned} I(x) = \log {\frac{1}{p(x)}} = -\log p(x) \end{aligned}$$

Mutual information: “Let X and Y be two random variables and x and y be their outcomes, respectively. The mutual information of X and Y measures the amount of reduction in uncertainty of the outcome x when the outcome y is known, or vice versa, and is defined as follows”^30^:

2 $$\begin{aligned} I(x;y)=\log {\frac{p(x\mid y)}{p(x)}} = -\log p(x)-(-p(x\mid y)) =I(x)-I(x\mid y) \end{aligned}$$

Let x, y represent the two nodes in a graph and $\Gamma (x), \Gamma (y)$ represent their set of neighbors. Also, common neighbors of x, y is represented by $O_{xy}$. So we can say that $O_{xy}=\Gamma (x)\cap \Gamma (y)$. Now for given node pair(x, y) and common neighbors $O_{xy}$; The likelihood score of node pair(x, y) can be computed by the following equation^31^.

3 $$\begin{aligned} S^{MI}_{xy}= -I(L^{1}_{xy}\mid O_{xy}) \end{aligned}$$

Here $I(L^{1}_{xy}\mid O_{xy})$ is the conditional self-information of the existence of an edge between node pair (x, y) when they have common neighbors $O_{xy}$. On analysis of self-information of the node-pair, it is found that the smaller $I(L^{1}_{xy}\mid O_{xy})$ is, the higher the probability of the existence of an edge. According to Eq. (2), we can derive the value of $I(L^{1}_{xy}\mid O_{xy})$ as follows:

4 $$\begin{aligned} I(L^{1}_{xy}\mid O_{xy})=I(L^{1}_{xy})-I(L^{1}_{xy};O_{xy}) \end{aligned}$$

where $I(L^{1}_{xy})$ represents the self-information of that node pair (x,y) that are already connected. $I(L^{1}_{xy};O_{xy})$ represents the mutual information between node pair (x,y) that has one link between them and the node pair’s common neighbors are known. Now let’s consider that the elements of $O_{xy}$ are independent of each other, then we can find the value of $I(L^{1}_{xy};O_{xy})$ as follows:

5 $$\begin{aligned} I(L^{1}_{xy};O_{xy}) =\sum _{z\in O_{xy}} I(L^{1}_{xy};z)=\sum I(L^{1}_{xy};z) \end{aligned}$$

Now $I(L^{1}_{xy};z)$ can be calculated by $I(L^1;z)$. $I(L^1;z)$ is defined as the mean mutual information over all node pairs connected to node z.

6 $$\begin{aligned} I(L^1;z) = \frac{1}{\mid \Gamma (z)\mid ( \mid \Gamma (z)\mid -1)} \sum _{\begin{array}{c} m \ne n \\ m,n \in \Gamma (z) \end{array}}I(L^{1}_{mn};z) \end{aligned}$$

Now we can find the value of $I(L^{1}_{mn};z)$ with the help of Eq. (2).

7 $$\begin{aligned} I(L^{1}_{mn};z) = I(L^{1}_{mn})-I(L^{1}_{mn}\mid z) \end{aligned}$$

Here $I(L^{1}_{mn})$ indicates the self-information of node pair (m, n) is connected. $I(L^{1}_{mn}\mid z)$ is simply the conditional information of that connected node pair (m, n) when node z is one of their common neighbors.

Now $I(L^{1}_{mn}\mid z)$ can be calculated by the clustering coefficient of node z and the clustering coefficient of z can be calculated as follows:

8 $$\begin{aligned} p(L^{1}_{mn}\mid z) =C_z= \frac{2*t_z}{d_z(d_z-1)} \end{aligned}$$

where $C_z$ represents the clustering coefficient of z. $t_z$ represents the number of triangles passing through node z and $d_z$ represents the degree of node z. Since we have value of $p(L^{1}_{mn}\mid z)$ we can easily find the value of $I(L^{1}_{mn}\mid z)$.

$p(L^{1}_{mn})$ can be computed with the help of $p(L^{0}_{mn})$. $L^{0}_{mn}$ represents the event that there is no edge that exists between node *m* and node *n*. It is considered here that no degree correlation exists. The value of $p(L^{0}_{mn})$ can be calculated with the help of path entropy.

9 $$\begin{aligned} p(L^{0}_{mn}) =\prod _{i=1}^{d_n} \frac{(T_l-d_m)-i+1}{T_l-i+1}=\frac{{}^{d_n}C_{T_l-d_m}}{{}^{d_n}C_{T_l}} \end{aligned}$$

Here $d_m$ and $d_n$ are the degree of node *m* and *n*, respectively. $T_l$ is the total no of edges in the graph. This formula is symmetric. Thus

10 $$\begin{aligned} p(L^{0}_{mn})=p(L^{0}_{nm}) \end{aligned}$$

So now $p(L^{1}_{mn})$ and $p(L^{1}_{nm})$ can be calculated as follows.

11 $$\begin{aligned} p(L^{1}_{nm})=p(L^{1}_{mn})=1- \frac{{}^{d_n}C_{T_l-d_m}}{{}^{d_n}C_{T_l}} \end{aligned}$$

With the help of Eq. (1) we can find the value of $I(L^{1}_{mn})$ and $I(L^{1}_{nm})$. Collecting these results, we can get the following things.

12 $$\begin{aligned}&I(L^{1}_{xy};z) \approx I(L^{1};z)=\frac{1}{\mid \Gamma (z)\mid ( \mid \Gamma (z)\mid -1)}\sum _{\begin{array}{c} m \ne n \\ m,n \in \Gamma (z) \end{array}}I(L^{1}_{mn})-I(L^{1}_{mn}\mid z) \end{aligned}$$

13 $$\begin{aligned}{\,}&\quad = \frac{1}{\mid \Gamma (z)\mid (\mid \Gamma (z)\mid -1)} \sum _{\begin{array}{c} m \ne n \\ m,n \in \Gamma (z) \end{array}}(-\log p(L^{1}_{mn})-(-\log p(L^{1}_{mn}\mid z))) \end{aligned}$$

14 $$\begin{aligned}{\,}&\quad = \frac{1}{\mid \Gamma (z)\mid (\mid \Gamma (z)\mid -1)} \sum _{\begin{array}{c} m \ne n \\ m,n \in \Gamma (z) \end{array}}\log \frac{{}^{d_n}C_{T_l}}{{}^{d_n}C_{T_l}-{}^{d_n}C_{T_l-d_m}} + \log \frac{2*t_z}{d_z(d_z-1)} \end{aligned}$$

So with the help of the previous derivation, we have

15 $$\begin{aligned} S^{MI}_{xy}= -I(L^{1}_{xy}\mid O_{xy})=\sum _{z\in O_{xy}} I(L^{1}_{xy};z)-I(L^{1}_{xy}) \end{aligned}$$

Here chances of the existence of an edge between node pair(x, y) are directly proportional to $S^{MI}_{xy}$. It simply means higher the $S^{MI}_{xy}$, the more likely the nodes will be connected.

## Methods

**Figure 2:**
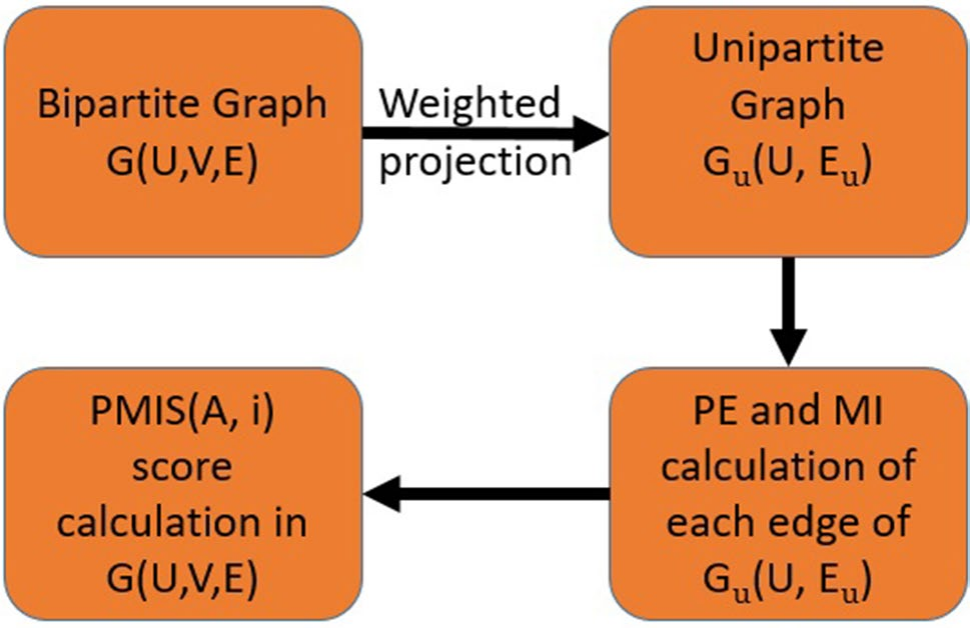
Link prediction process.

In this section, we are presenting a novel algorithm for link prediction in the bipartite networks based on potential energy and mutual information. The proposed algorithm majorly works on the four concepts projection, potential energy mutual information and PMIS. Figure 2 briefly describes the process for PMIS score calculation.

In this work we define Potential Energy in the context of graphs. We assumed that a pair of nodes act as an object and that the product of the degree of nodes of the graph can represent mass. The gravitational acceleration g can be represented by the sum of the clustering coefficient of common neighbor between two nodes. For a given pair of nodes, sum of the clustering coefficient of common neighbor will be constant, but for a different pair of nodes the value of the sum of the clustering coefficient of common neighbors will be different. Also, we replaced distance (h) by inverse of the shortest distance (sd) between the pair of nodes.

*Potential energy* PE(A, B) represents the potential energy between nodes A and B. In the context of a graph, this can be defined as the product of three terms; the product of the degree of nodes, the sum of the clustering coefficient of common neighbors and the shortest distance between nodes.

16 $$\begin{aligned} PE(A, B)=(d_Ad_B)\left( \sum _{\begin{array}{c} \\ z \in \Gamma (A)\cap \Gamma (B) \end{array}}cl_z\right) \left( \frac{1}{sd(A, B)} \right) \end{aligned}$$

where $d_A$, $d_B$ are degree of nodes A and B. $cl_z$ is clustering coefficient of z $\forall z\ne \phi$. *sd*(*A*, *B*) represents the shortest distance between node A and B. When $z = \phi$; that is no common neighbor between node pair(A, B), and is such cases the value of $cl_z$ will be .1(constant).

**Figure 3:**
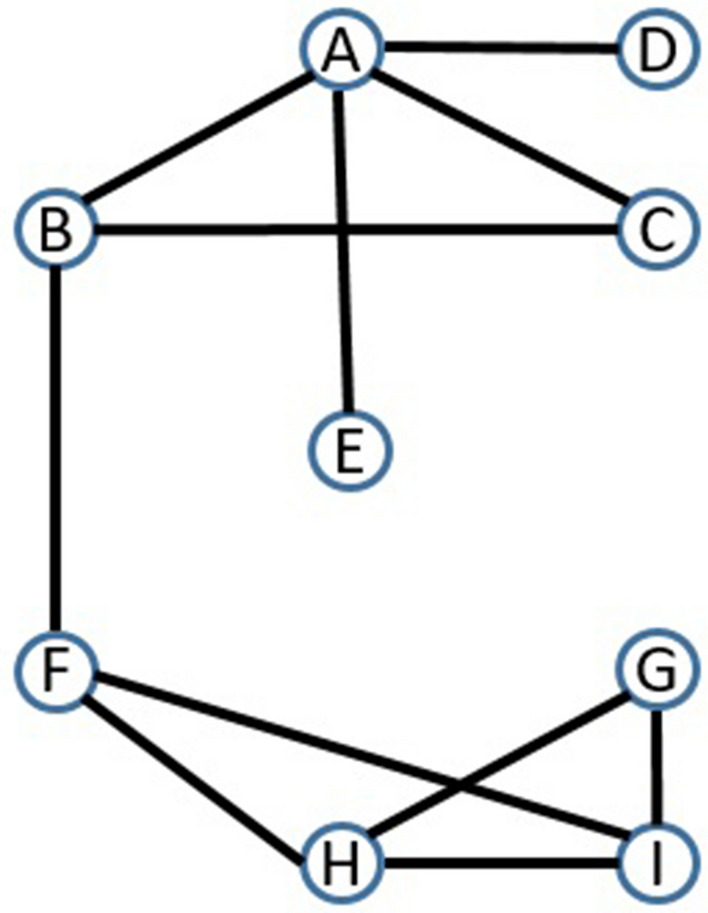
Graph G2.

**Table 1:** Potential energy computation for some missing edges in Graph G2.

Edge (*x*, *y*)	Product of the degree of nodes	The sum of the clustering coefficient of common neighbors	Inverse of the shortest distance between nodes.	*PE*(*x*, *y*)
(B, E)	3	.1666	.50	.25
(C, E)	2	.1666	.50	.16
(E, G)	2	.1	.20	.04
(E, F)	3	.1	.33	.1

To simplify the understanding of PE, we illustrate it with an example. In Fig. 3, the values of PE between node pairs(B, E), (C, E) and (E, G) are .25, .166, and .04 respectively. We used Eq. (16) for PE calculation and Eq. (8) for calculation of the clustering coefficient of the node. Here PE of node pair(B, E) is greater than (C, E). So it shows that Node pair(B, E) is more likely to be connected than node pair(C, E). PE can distinguish node pairs even if they have no common neighbour. For example, PE of node pair(E, F) and (E, G) are .1 and .04 respectively. So this shows that node pair(E, F) is more likely to be connected than node pair(E, G). The value of the clustering coefficient, product of the degree of nodes and the shortest distance of illustrative examples are given in Table 1.

The potential energy of the network incorporates three different properties in itself. One part of potential energy talks about the product of the degree of vertices. Higher the values of product, higher the PE. If we think from the social networks point of view, vertex having a higher degree has always high likelihood to connect with another vertex. For example, a new person joining Twitter has a higher likelihood to follow a celebrity than not so popular people. Because the degree of celebrities is usually higher. The second part of PE is the clustering coefficient. The significance of the clustering coefficient in social networks is that a person tends to have friends who are also friends with each other. It is very closely related to triadic closure. Triadic closure plays a very important role in link prediction. So the clustering coefficient has the inherent properties of the link prediction. And the third part of PE is the shortest distance between vertices. This is another important feature of networks. Kleinberg found that most of the nodes in the social networks are connected with a very short distance^32^. Distance between nodes has inverse effect on link prediction. This is also related to the small world phenomenon. In real social networks if the distance between two people is smaller that means they have a higher chance of becoming friends in the future. Illustrative example and results also show the effectiveness of PE.

### Algorithm framework

The proposed algorithm initially takes a bipartite graph *G*(*U*, *V*, *E*) as input and using weighted projection transforms it into a unipartite graph $G_u(U,E_u)$. In the unipartite graph $G_u(U, E_u)$, the proposed algorithm computes PE and MI for each node pair using Eqs. (15) and (16) respectively. Then algorithm calculates weight of edge(pattern) of unipartite graph using Eq. (17). After the calculation of the weight of the pattern, the proposed algorithm uses Eq. (18) and compute the PMIS score for each node pair of bipartite graph *G*(*U*, *V*, *E*).

**Figure 4:**
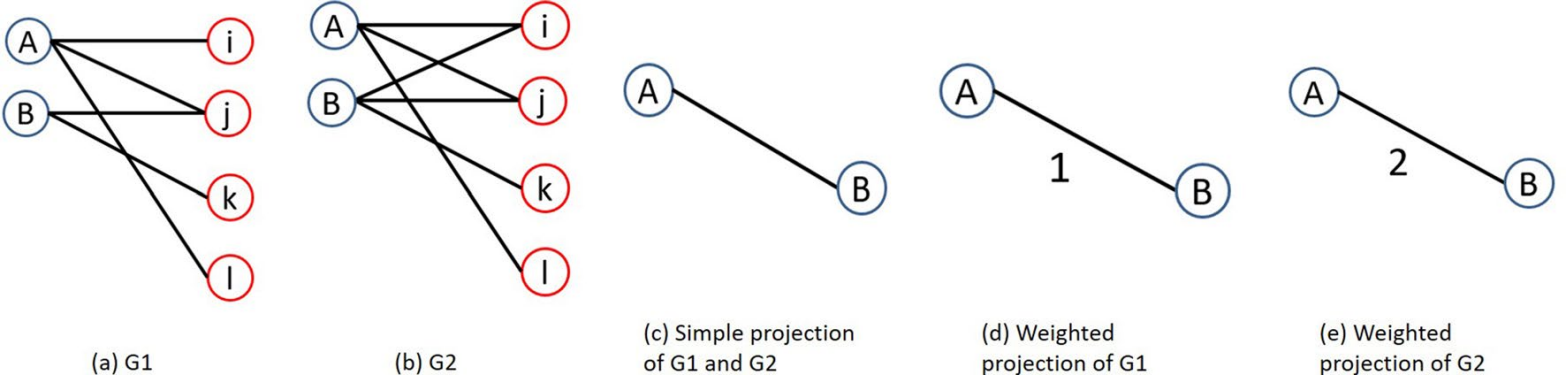
Projection of bipartite graph.

The proposed algorithm takes weighted projection instead of simple projection. The main reason of taking weighted projection is that in simple projection we lose the topological information of the original bipartite graph. To keep such information we use weighted projection. For example, two bipartite graphs in Fig. 4a,b. are different but their simple projection is the same. Figure 4c shows the simple projection of bipartite graph Fig. 4a,b. But if we take weighted projection then we get two different projected graph Fig. 4d,e. In Fig. 4d edge weight is 1 because only one common neighbor is present in the bipartite graph. But in Fig. 4e edge weight is 2 because there are 2 common neighbors in the bipartite graph. WP(A, B) represents the weight of the edge of the projected graph and we calculate it as follows.

$$\begin{aligned} WP(A,B )=\mid \Gamma (A)\cap \Gamma (B) \mid , (A,B) \in U \end{aligned}$$

Since we have a projected graph of the original bipartite graph, so by using Eq. (15) we can estimate the $S^{MI}_{AB}$ for each node pair of the projected graph. Or we can say in another way that for every pattern we have $S^{MI}_{AB}$.

The weight of the edge in the projected graph plays a very important role in link prediction. This weight is nothing but the weight of pattern, which we have already defined. So now we can say that for each pattern we have three values PE(A, B), WP(A, B) and $S^{MI}_{AB}$. These values declare the importance of pattern.

#### **Definition 0.1**

Total Weight of pattern, $W_t(A, B)$ represents the sum of all types of weight of the pattern (edge A, B in projected graph) and is defined as follows:

17 $$\begin{aligned} W_t(A,B)=PE(A, B) + S^{MI}_{AB}+WP(A,B) \end{aligned}$$

#### **Definition 0.2**

Potential Energy-Mutual Information based similarity metrics(PMIS): PMIS is the sum of the weight of all pattern covered by a node pair.

If (A, i) is a node pair in bipartite graph $G=(U, V, E)$ then PMIS can also be defined as

18 $$\begin{aligned} PMIS(A,i)=\sum _{\{A,B\}\in \Gamma (A,i)} W(A,B) \end{aligned}$$

Here *W*(*A*, *B*) is the weight of the pattern $\{A, B\}$. And $\Gamma (A, i)$ is the set of patterns covered by node pair (*A*, *i*). We can find the value of $\Gamma (A, i)$ as follows:

$$\begin{aligned} \Gamma (A,i)=\{(A, B)\mid B \in N_u (A) \cap N (i) \} \end{aligned}$$

So it is clear that every node pair in the bipartite graph cover some pattern. And each pattern has some weight. Therefore PMIS computes the sum of the weight of all pattern covered by a node pair. Here we are using PMIS value as the final link prediction score. Higher PMIS value indicates higher likelihood of the existence of an edge.

*Complexity analysis* The time complexity of proposed algorithm for a given bipartite graph, *G*(*U*, *V*, *E*) with two vertex sets, *U* and *V* is $O(|U|^3)$, where $|V|<|U|$. The algorithm has three steps. Bipartite graph is converted into unipartite graph in the first step with time complexity $O(|V|d^2)$. Here, d denotes the maximum vertex degree of given bipartite graph. Since d is constant, the complexity of first step can be rewritten as *O*(|*V*|). The second step of the algorithm computes PE and MI and the complexity of this step is $O(|V|^3)$. PMIS is computed in the last step with $O(|V|d^3)$ time complexity. Therefore, total time complexity of the algorithm is $O(|V|^3)$. Moreover, the complexity improves to *O*(|*V*|) when algorithm focuses on candidate node pair instead of focusing on each node pair. This complexity is better than $O((|U|+|V|)^3)$ time complexity of Katz algorithm.

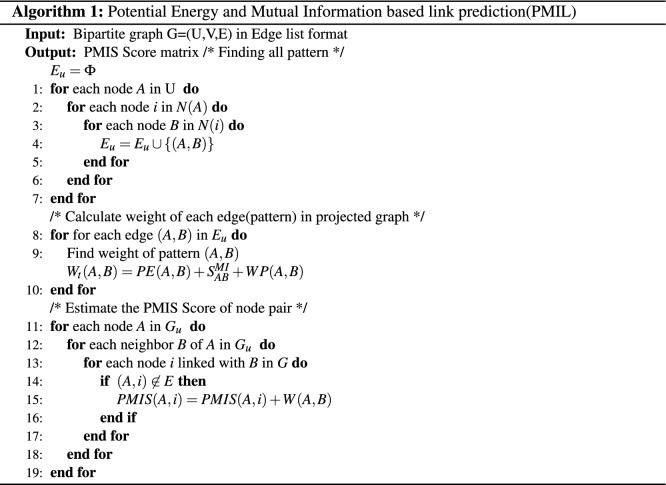



## Results and discussion

All experiments were conducted on a Linux server with an Intel XeonE5-2630 v3 2.40 GHz CPU and 64GB memory running CentOS 7.4-1708. We implemented PMIL and all other algorithms in Python 3.7.0. In all experiments majorly used networkx, pandas, sklearn, numpy, and matplotlib library.

### Evaluation metrics

Two standard metrics are generally used to quantify the accuracy of any prediction algorithms, one is area under the receiver operating characteristic curve (AUC)^33^ and another is Precision^34^. We have performed an extensive experiment and used four metrics to test the performance of the proposed algorithm. Following are the names of four metrics used for the performance evaluation.

*AUC* AUC value can be defined as the probability that a randomly chosen missing link (i.e., a link in $$E^p$$ ) is given a higher score than a randomly chosen nonexistent link (i.e., a link in $$U-E$$). Here if suppose among $$N^t$$ independent comparisons, $$ N^h, $$ times the existing edge having a higher score and $$ N^s $$ times they have the same score,then AUC score can be calculated by following equation.

19 $$\begin{aligned} AUC = \frac{\left( {N^h} + .5N^s\right) }{N^t} \end{aligned}$$

In general, a larger AUC value indicates high performance. The AUC value of the ideal result is 1.0.

*Precision* Precision is defined as the ratio of relevant items selected to the number of items selected. After sorting the scores, if there are $L_r$ links belonging to the test set among top-*L* candidate links, then Precision is obtained by the following equation.

20 $$\begin{aligned} Precision=\frac{L_r}{L} \end{aligned}$$

*Prediction-Power (PP)* This metric is used to check the deviation from the mean random-predictor performance^20^. PP is computed as follows:

21 $$\begin{aligned} PP=\log _{10} \frac{Precision_{Prediction Technique}}{Precision_{Random}} \end{aligned}$$

where $Precision_{Random}$ is the result of random-predictor. And we can compute it by $\frac{L}{|U||V|-(E-L)}$.

*Precision@K* It is the fraction of correct predictions in top *k* predictions^34^. In our paper, we computed Precision@10, Precision@20 and Precision@50. Precision@10 means precision at the top 10 position in the ranking result. The higher the values of the metrics are, the better the algorithm is.

To evaluate the performance of our model, we used the K-fold Cross-Validation. K-Fold CV is a technique in which a given data set is split into a *K* number of sections/folds. Each time one subset is chosen as a probe set and the rest $K-1$ used as training set. Here we have taken the value of K is 10.

### Datasets

*Datasets* We used ten real-world datasets to test the performance of the proposed algorithm. These ten datasets are the following: ** (1) MovieLens (ML)**^35^ dataset contains 100,000 ratings from 943 users on 1682 movies. **(2) Enzyme (EN)**^36^ is a biological network of drugs and enzymes proteins. It contain 445 drugs nodes, 664 proteins nodes and 2926 drug–target interactions. **(3) Southern Women network dataset (SWN)**^37^ represents 18 women who participated in 14 social events. **(4) Corporate Leadership bipartite graph dataset (CL)**^38^ contains the person’s name and company name. **(5) Club membership dataset (CM)**^39^ contains participation data of corporate officials in social associations. **(6) Ionchannels (IC)**^36^ is biological network of drug and ionchanel proteins. **(7) Country-organization (C2O)** is global network of country and various organization **(8) Drug target (Drug)**^40^ is a chemical network of drug target interaction. **(9) G-protein coupled receptors (GPC)**^36^ is biological network. **(10) Malaria(mal)**^41^ is a genetic network. Table 2 shows the topological features of all the datasets.

*Results* To test the strength of the PMIL algorithm, we performed extensive experiments on ten different real-world datasets and compared it with eleven baseline link prediction techniques. Since our proposed algorithm comes under the similarity-based technique, so for the comparison purpose we considered mainly similarity-based algorithm. The baseline link prediction techniques include Common Neighbors (CN), Jaccard Coefficient (JC), Preferential attachment (PA), Cannistraci–Alanis–Ravasi (CAR), Cannistraci–Jaccard (CJC), Cannistraci–Adamic–Adar (CAA), Cannistraci resource allocation (CRA), Nonnegative Matrix Factorization (NMF), Cosine (CS), Potential Link prediction (PLP) and Bipartite projection via Random-walk (BPR)^10,21,42^. Out of these 11 baseline algorithms, three works on node neighbourhood mechanism, four works on LCP mechanism, three works on projection mechanism and one works on latent feature mechanism.

**Figure 5:**
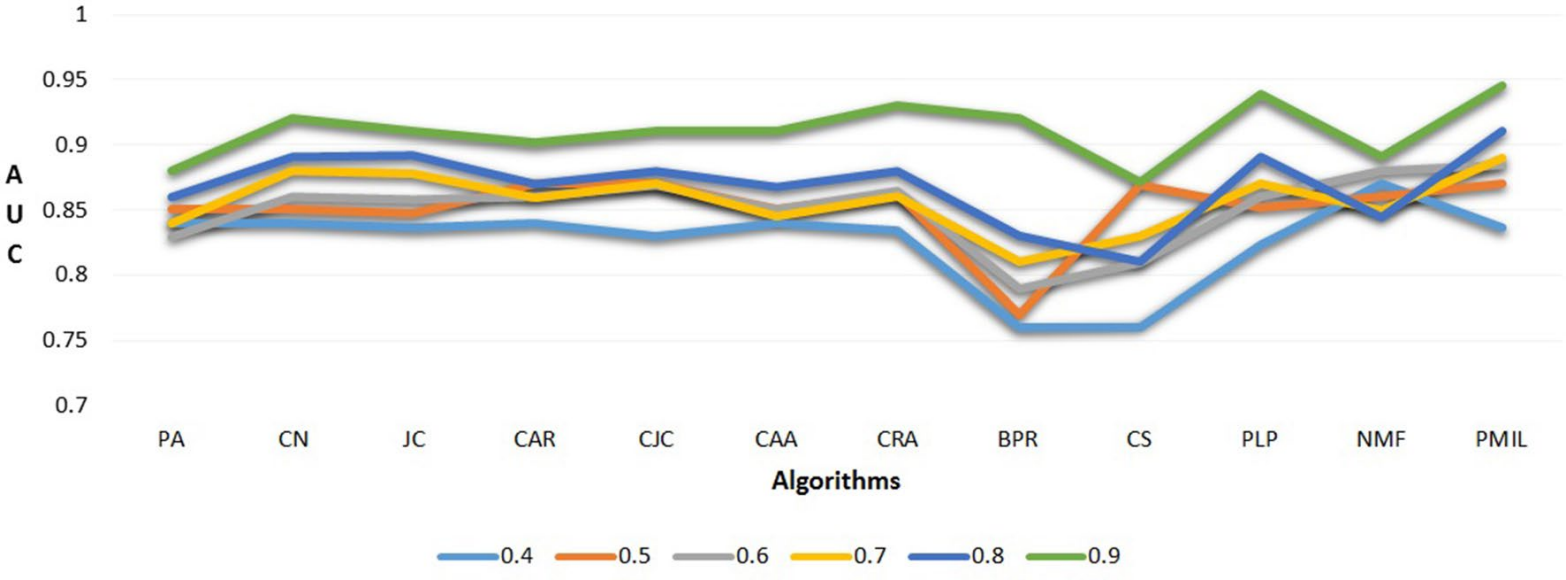
AUC values for baseline algorithms where the size of the training set varies from 0.4 to 0.9 tested on Drug dataset.

The AUC and Precision values of the proposed algorithm and other baseline algorithms are listed in Tables 3 and 4 respectively. In these tables, each row represents the method used in the experiment and each column represents the datasets. The largest value in each column is represented in bold text. In each of these 10 datasets, the test set contains 10% edges and training set contains 90% edges. Table 3 shows the proposed PMIL algorithm outperforms the ten baseline link prediction algorithms on seven datasets for AUC values. But on the CL and IC dataset winner is CAA and PLP respectively. Interestingly, the value of AUC for CAA and CRA are same on C2O dataset. Since the AUC value of the PMIL algorithm is better so if we draw ROC curve by plotting true-positive rates (TPR) versus false-positive rates (FPR) for varying L values then the total area under the ROC-curve (AUC) will be more. Thus it indicates the better prediction result quality, where L is the list of top links as predicted links. The results in Table 4 demonstrate that PMIL algorithm gives best precision values on six datasets (ML, EN, SWN, CM, Mal, GPC). However, CAA and PLP are winners for CL and IC datasets respectively; and performance of BPR algorithm is best on both C2O and Drug dataset based on the Precision value. Figure 5 shows the effects of the size of the training set on AUC for Drug dataset. We experimented by changing the size of the training set from 40% to 90%. It can be observed from Fig. 5 that on increasing the size of the training set to 0.9, all baseline algorithms as well as PMIL gives better AUC scores.

**Table 2:** Statistics of the dataset, where ML(MovieLens), EN (Enzyme), SWN (Southern Women Network), CL (Corporate Leadership), CM (Club Membership), IC (Ion channels), C2O (Country-Organizations), Mal (Malaria), GPC (G-protein coupled receptors) and Drug (Drug Target) are the names of the datasets and Data Types represent the domain of the datasets.

Name of dataset	Number of nodes	Number of edges	Average degree	Dataset types
ML	2625	85,250	32.48	Entertainment
EN	1109	2926	5.2	Biological
SWN	32	89	5.5	Social network
CL	64	99	4.5	Management
CM	65	95	4.7	Social network
IC	414	1476	3.5	Biological
C2O	295	12,170	41.2	Global network
Mal	1103	2965	2.6	Genetic network
GPC	318	635	2	Biological
Drug	350	454	1.3	Chemical network

**Table 3:** AUC comparison results on ten datasets (ML, EN, SWN, CL, CM, IC, C2O, Mal, GPC and Drug).

Dataset	ML	EN	SWN	CL	CM	IC	C2O	Mal	GPC	Drug
PA	.881	.788	.648	.773	.764	.823	.901	.591	.720	.880
CN	.871	.851	.730	.811	.801	.910	.990	.901	.812	.920
JC	.791	.880	.663	.821	.798	.850	.950	.901	.821	.910
CAR	.912	.867	.726	.940	.906	.916	.990	.910	.811	.901
CJC	.882	.867	.762	.960	.940	.925	.990	.910	.831	.910
CAA	.910	.851	.760	**.968**	.950	.942	**1.00**	.920	.831	.910
CRA	.921	.890	.772	.945	.955	.931	**1.00**	.910	.821	.930
BPR	.911	.891	.742	.943	.959	.920	.990	.901	.840	.920
CS	.831	.836	.761	.775	.882	.835	.960	.821	.801	.871
PLP	.930	.889	.936	.905	.960	**.945**	.965	.906	.849	.938
NMF	.891	.761	.692	.854	.846	.850	.990	.861	.702	.890
PMIL	** .945**	**.901**	**.945**	.940	**.982**	.938	.971	**.921**	** .867**	**.945**

Each dataset is divided into training set (90%) and test set (10%) and results are computed by averaging over 1000 runs.

**Table 4:** Precision comparison results on ten datasets averaged over 1000 runs.

Dataset	ML	EN	SWN	CL	CM	IC	C2O	Mal	GPC	Drug
PA	.153	.023	.122	.110	.157	.036	.871	.022	.081	.313
CN	.141	.370	.141	.210	.202	.230	.871	.192	.310	.610
JC	.001	.031	.021	.042	.036	.021	.601	.250	.012	.383
CAR	.177	.507	.188	.189	.202	.432	.871	.191	.332	.601
CJC	.184	.496	.188	.217	.231	.494	.871	.232	.361	.191
CAA	.181	.502	.122	** .662**	.621	.531	.870	.191	.320	.591
CRA	.181	.651	.210	.612	.631	.560	.880	.251	.373	.631
BPR	.181	.501	.162	.620	.641	.442	** .931**	.253	.271	**.680**
CS	.120	.330	.163	.165	.455	.349	.661	.142	.201	.491
PLP	.191	.491	.410	.210	.620	**.612**	.631	.221	.283	.301
NMF	.001	.001	.031	.022	.031	.011	.001	.001	.012	.021
PMIL	**.210**	** .661**	** .441**	.205	** .651**	.581	.601	**.261**	** .401**	.310

**Figure 6:**
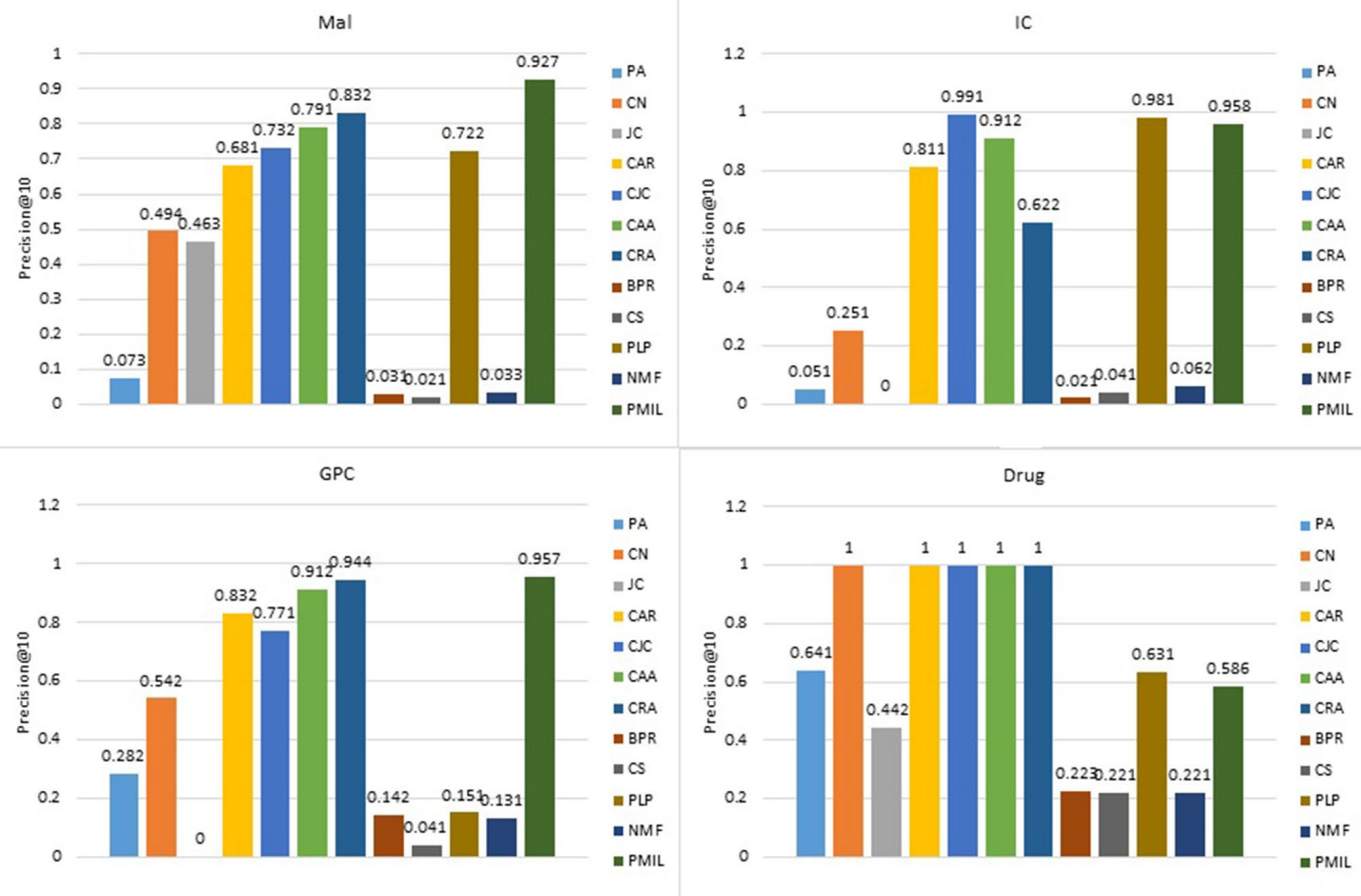
Comparison of precision@10 on the four datasets.

**Figure 7:**
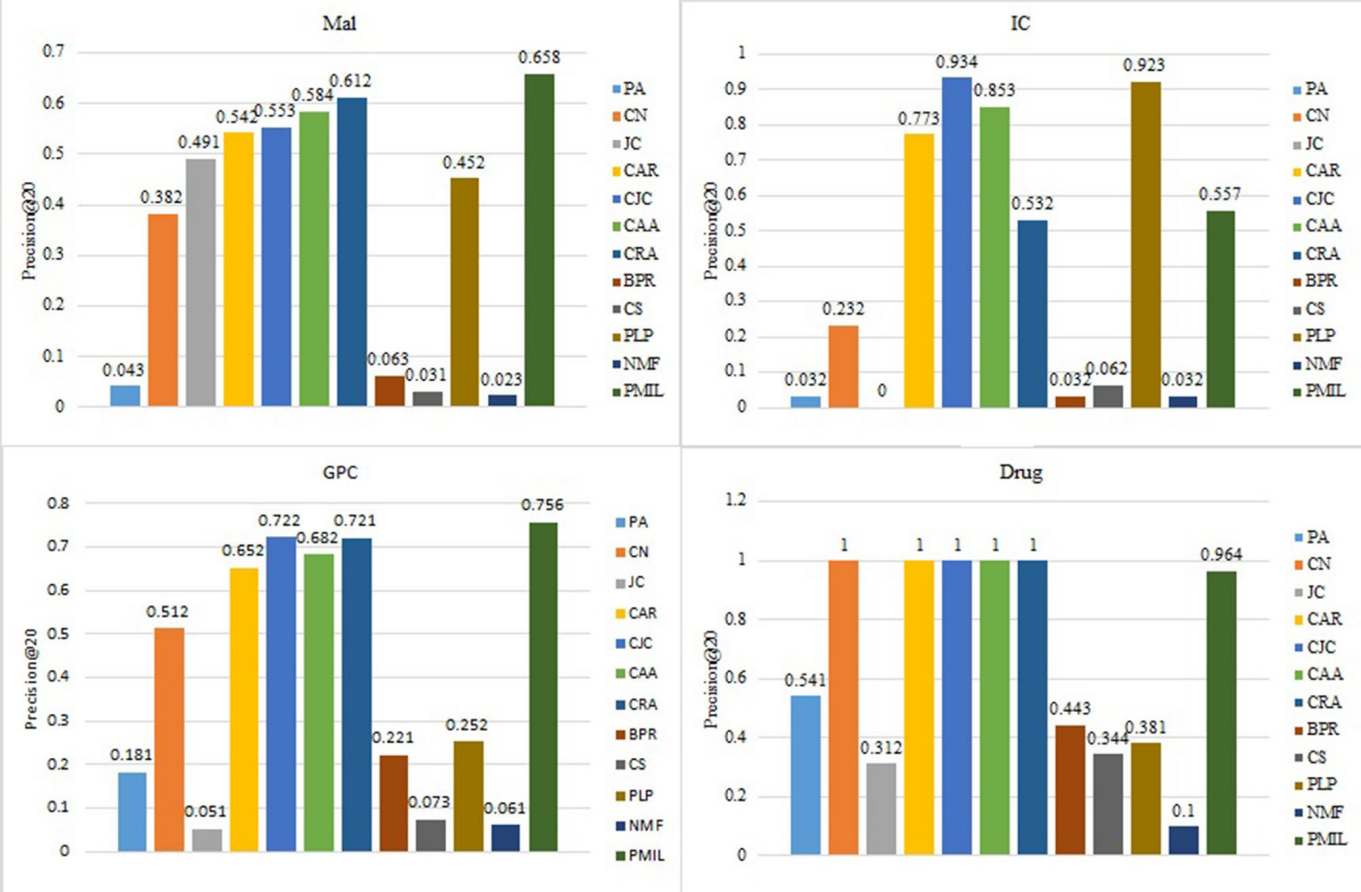
Comparison of precision@20 on the four datasets.

**Figure 8:**
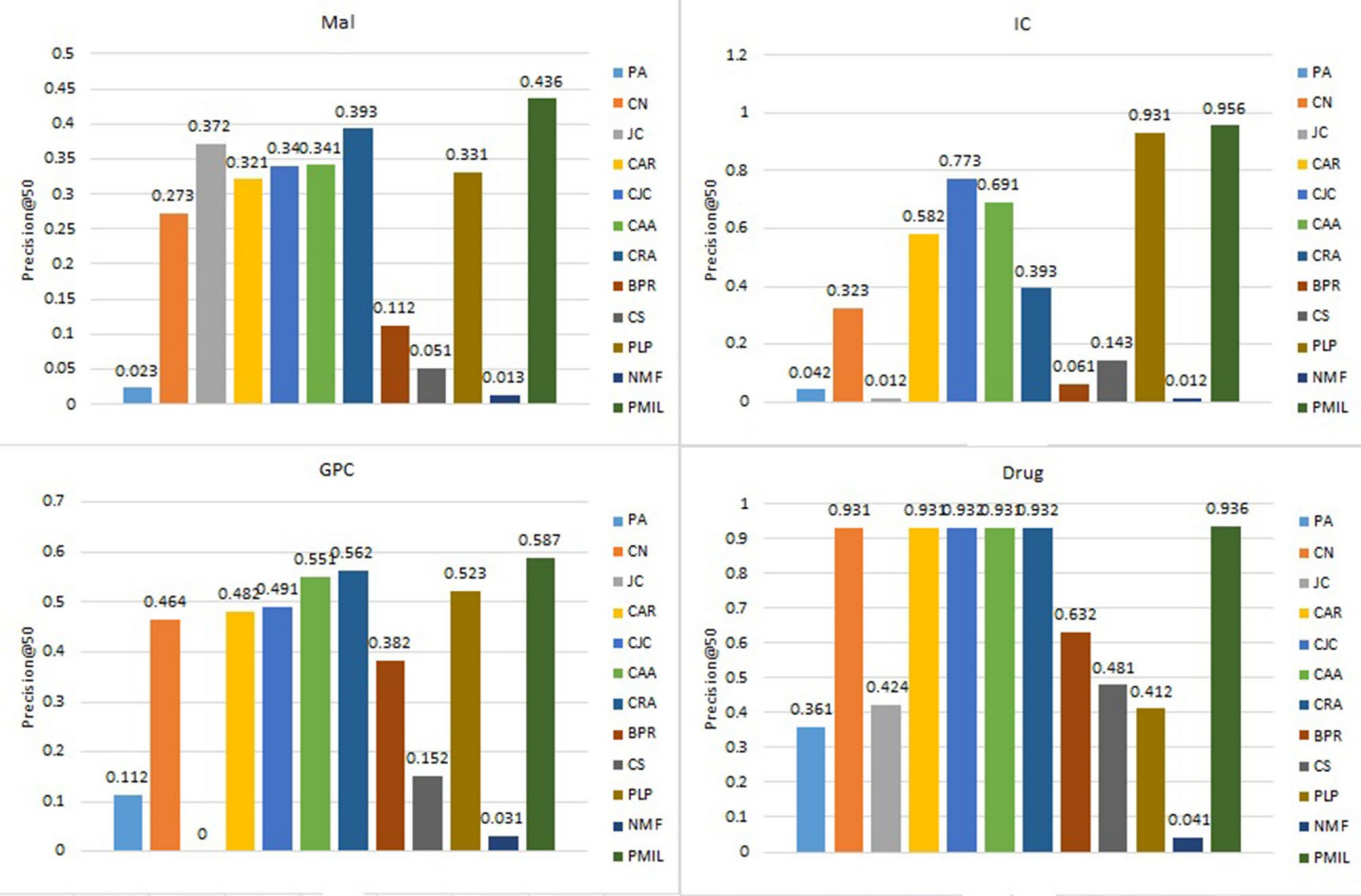
Comparison of precision@50 on the four datasets.

**Figure 9:**
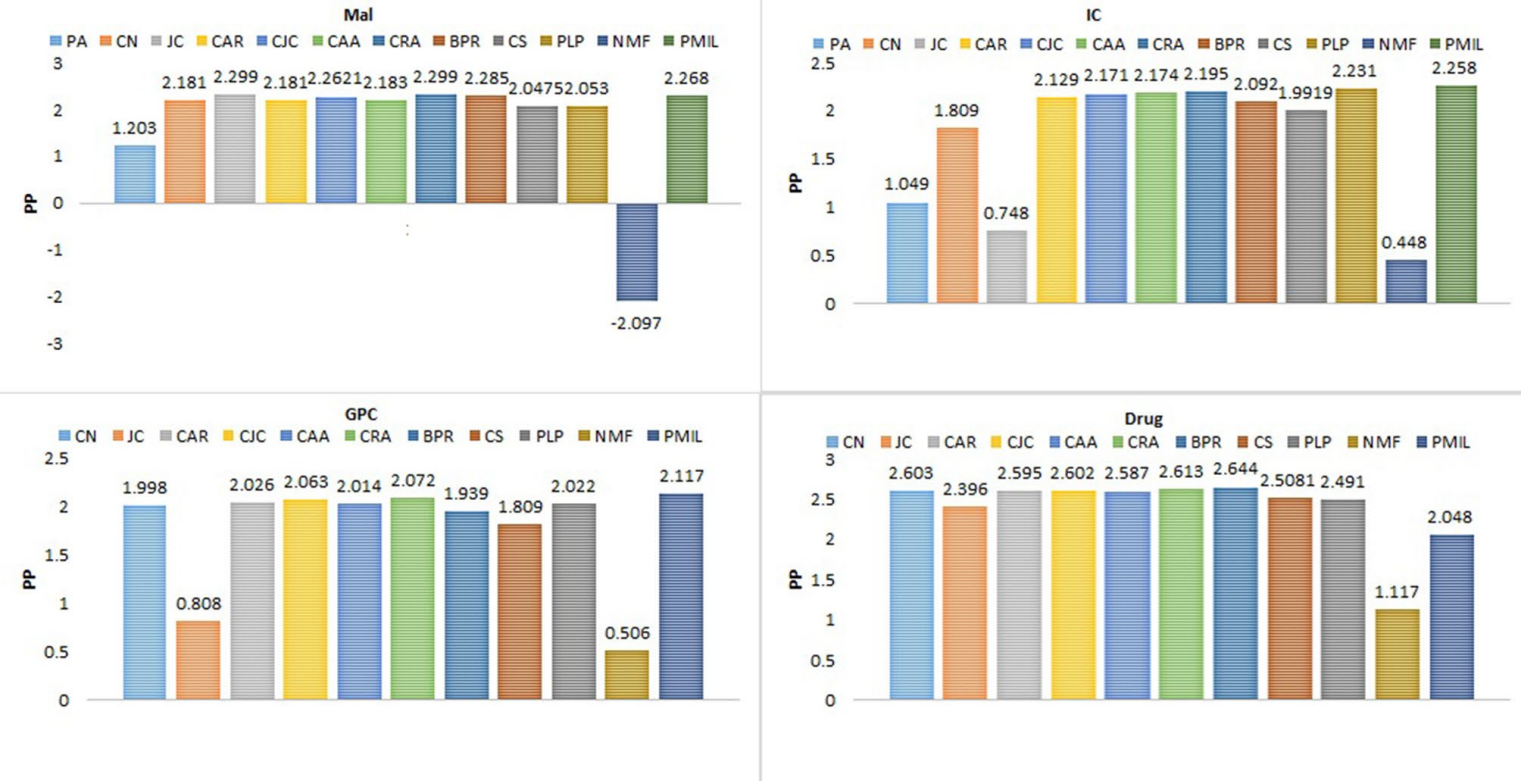
Comparison of prediction-power on the four datasets.

Figures 6, 7 and 8 describes the precision@K for different values of K. To keep the image clear, we presented the result of twelve algorithms on four datasets. In Fig. 6 we presented the value of precision@10 for the Mal, IC, GPC and Drug datasets. For the IC dataset, CJC and PLP have better performance than PMIL. But our proposed algorithm shows a better result than all eleven baseline algorithms on the Mal and GPC dataset. This improvement is very useful in the recommender system. Especially in E-commerce, where we are interested to show only the top 10 or top 20 or top K results among the best results to the customer.

Figures 7 and 8 show the precision@20 and precision@50 values respectively. The proposed algorithm shows the highest value of Precision@20 for Mal and GPC dataset. The proposed algorithm gives the best result for precision@50 on Mal, IC, GPC and Drug dataset. Interestingly, all the algorithms based on LCP-theory (CAR, CJC, CAA, CRA) and our proposed algorithm show almost similar results for drug dataset.

Figure 9 describes the prediction-power of all twelve algorithms over the four datasets. For the IC and GPC datasets, PMIL secured the first position. And for Mal datasets, JC and CRA both are at first position, whereas in case of the Drug dataset winner is BPR. PP metric is very useful when we are interested to find which algorithms have a minimum or maximum deviation from the mean random-predictor. So basically it characterises the deviation of the algorithm from the randomness.

## Conclusion

In this paper, we introduced a novel approach for link prediction in bipartite networks which is based on the concepts of potential energy and mutual information. The performance of the proposed algorithm is evaluated on ten datasets under different classes and compared with eleven baseline predictors on the basis of AUC, Precision, Prediction-Power and Precision@K. We used AUC for evaluation of PMIL, in which we assume that all the links in the networks are independent of each other. However in the real-world networks, links may or may not be independent of each other. PMIL algorithm showed best performance on seven out of the ten datasets used based on AUC score and was reasonably close for the three remaining datasets. There is a possibility that the structural properties of non-social network graphs effect the performance; thus it be interesting to study the structural properties, such as the community structure, of different datasets and compare any significant differences between social networks and other datasets. In social networks a connection implies that there was an interaction in the past, but the complete information about the connections may be missing. The other datasets may be noisy and an example could be biological datasets, where the connections are identified with the help of experiments that are not always accurate. Thus, expanding PMIL such that it is capable of link prediction in weighted bipartite graphs is another future direction of research, where weight could be the probability of the occurrence of edge. There are many applications of link prediction, such as recommender systems, community detection, finding hidden relationships, etc. Thus, it would be interesting to explore the future directions and design more efficient algorithms for link prediction in bipartite networks.
